# Intraoperative Analgesiesteuerung: Analgesie Nociception Index (ANI) vs. „standard care“ bei Hysterektomien unter Sevoflurannarkose: Eine randomisierte kontrollierte Einfachblindstudie zu intraoperativem Opioidverbrauch, postoperativen Schmerzen und Patientenzufriedenheit

**DOI:** 10.1007/s00101-023-01288-y

**Published:** 2023-06-07

**Authors:** A. M. Kunst, H. Wulf, B. Stegemann, A. Fiehn

**Affiliations:** 1 Klinik für Anästhesie und Intensivmedizin, Uniklinik der Philipps-Universität Marburg, Marburg, Deutschland; 2 Klinik für Anästhesiologie und Intensivmedizin, Agaplesion Diakonie Kliniken Kassel, Kassel, Deutschland; 3 Herkulesstraße 34, 34119 Kassel, Deutschland; 4 Zentrum für klinische Forschung Kassel, Kassel, Deutschland

**Keywords:** Analgesie, Nozizeption-Monitoring, Fentanylverbrauch, Opioidinduzierte Nebenwirkungen, Schmerzscore, Analgesia, Nociception monitoring, Fentanyl consumption, Opioid induced side-effects, Pain score

## Abstract

**Hintergrund:**

Während zu klinischer Überwachung und Kontrolle von Hypnose und Muskelrelaxation validierte Methoden im Anästhesiealltag existieren, basiert die Einschätzung der Analgesie immer noch überwiegend auf der Interpretation klinischer Vitalparameter. In der vorliegenden klinischen Studie wurde untersucht, ob die Verwendung eines „Nozizeption-Monitors“ zur Erfassung des intraoperativen Analgetikabedarfs der bisherigen Analyse der Vitalparameter überlegen ist. Zur quantitativen Erfassung der Analgesie wurde der Analgesia Nociception Index (ANI; Fa. MDoloris, Lille, France) verwendet. Dieser beruht auf der Analyse der atemabhängigen Herzfrequenzvariabilität.

**Methode:**

Es handelt sich um eine klinische prospektive randomisierte kontrollierte Einfachblindstudie an 110 Patientinnen, welche sich einer laparoskopischen Hysterektomie in balancierter Anästhesie in der Agaplesion Diakonie Kliniken Kassel unterzogen.

Bei der Interventionsgruppe (ANI) erfolgte die intraoperative Analgetikagabe unter Verwendung des ANI-Monitors, wohingegen in der Vergleichsgruppe (VER) die Analgetikadosierung nach bisherigen klinischen Parametern (Vitalparameter, intraoperative Abwehrbewegungen) erfolgte. Anschließend wurden die Gruppen im Hinblick auf intraoperativen Opioidverbrauch (Fentanyl), postoperative Schmerzen und opioidinduzierte Nebenwirkungen sowie Patientenzufriedenheit am 3. postoperativen Tag verglichen.

**Ergebnisse:**

Insgesamt wurden 101 Patientinnen analysiert. Unsere Beobachtungen ergaben einen höheren durchschnittlichen intraoperativen Fentanylverbrauch in der Interventionsgruppe, bedingt durch eine signifikant höhere Anzahl an Einzelgaben (0,54 vs. 0,44 mg, *p* < 0,001). Bezüglich der weiteren Beobachtungspunkte gab es kaum Unterschiede zwischen den Gruppen. Bei der Patientenbefragung am 3. postoperativen Tag ergab sich ein Unterschied bezüglich einer höheren subjektiv geschilderten Vigilanzminderung in der ANI-Gruppe, nicht jedoch anderer Nebenwirkungen oder der Zufriedenheit mit der Schmerztherapie insgesamt.

**Schlussfolgerung:**

Eine Optimierung der Schmerztherapie durch intraoperative Zuhilfenahme des Analgesia-Nociception-Index(ANI)-Monitors bei Hysterektomiepatientinnen unter balancierter Anästhesie mit Sevofluran und Fentanyl konnte demzufolge nicht nachgewiesen werden.

**Zusatzmaterial online:**

Die Online-Version dieses Beitrags (10.1007/s00101-023-01288-y) enthält den zugrunde liegenden Fragebogen.

## Abstract (en)

**Background:**

General anesthesia leads to different effects depending on the drugs used (hypnosis, analgesia, muscle relaxation). While there are validated methods for clinical monitoring and control of hypnosis and muscle relaxation in routine anesthesia, the assessment of analgesia is still mainly based on the interpretation of clinical vital parameters, such as heart rate, blood pressure, perspiration or intraoperative movements of the patient. The present clinical study investigated whether the use of a so-called nociception monitor to record the intraoperative need for analgesics is superior to the previous analysis of vital parameters. The analgesia nociception index (ANI; MDoloris, Lille, France) was used, which is one of several nociception monitors currently on the market for recording the sympathicovagal balance. In the case of the ANI, the measurement is based on the analysis of the heart rate variability (HRV) as a function of breathing. The index is given in the form of a dimensionless score between 0 and 100, where 0 stands for a lack of parasympathetic activity and 100 for a very strong parasympathetic activity. According to the manufacturer, a value between 50–70 under anesthesia corresponds to sufficient intraoperative analgesia.

**Methods:**

This was a clinical prospective randomized study in which 110 patients who underwent laparoscopic hysterectomy under balanced anesthesia using propofol, fentanyl and atracurium for induction and sevoflurane and fentanyl for anesthesia maintenance, were divided into two groups. In the intervention group (ANI group), during the operation analgesics were administered with the aid of the ANI monitor (bolus of 0.1 mg fentanyl if the ANI was < 50), whereas in the comparison group analgesics were administered according to previous clinical parameters (vital parameters, intraoperative defensive movements). The groups were then compared with respect to intraoperative fentanyl consumption (primary outcome), postoperative pain and opioid-induced side effects (measured by the numeric rating scale [NRS]) as well as patient satisfaction on postoperative day 3 (secondary outcome).

**Results:**

The observations showed a higher total intraoperative consumption of fentanyl in the intervention group, due to a significantly higher number of individual doses (0.54 vs. 0.44 mg, *p* < 0.001). With respect to the other observation points, there were hardly any differences between the groups, neither with respect to the pain score nor the side effects in the recovery room. At most there was a trend towards a slightly lower pain score at the first measurement point in the recovery room (NRS at 15 min). In the patient survey on the postoperative day 3, there was a difference with respect to the subjectively described reduction in vigilance in the ANI group, but not other side effects or overall satisfaction with the pain therapy.

**Conclusion:**

In this group of patients the additional use of the ANI monitor for intraoperative control of analgesia led to an increased consumption of fentanyl compared to the comparison group, without having an impact on the postoperative pain score, opioid-induced side effects or patient satisfaction. An optimization of pain therapy through intraoperative use of the ANI monitoring in hysterectomy patients under balanced anesthesia with sevoflurane and fentanyl could therefore not be proven. Transferability of the results to a much older and/or sicker patient collective remains questionable.

Die intraoperative Nozizeptionsmessung mit dem Ziel der bedarfsgerechten intraoperativen Analgetikadosierung zur Vermeidung einer Unter- bzw. Überdosierung von Opioiden und deren Folgen ist ein aktuelles Feld der Anästhesieforschung.

Es befinden sich bereits mehrere kommerzielle Nozizeption-Monitore auf dem Markt, welche durch die Erhebung verschiedener Parameter eine Darstellung der Analgesie-Nozizeption-Balance versprechen.

Einer der ersten dieser Monitore ist der „Analgesia Nociception Index (ANI)“, welcher auf einer kontinuierlichen Analyse der Herzfrequenzvariabilität beruht. In unserer klinisch-randomisierten Studie haben wir die Verwendung des ANI zur intraoperativen Dosierung von Analgetika mit dem bisherigen klinischen Standard in Bezug auf den intraoperativen Analgetikabedarf, postoperative Schmerzen und Patientenzufriedenheit verglichen.

## Hintergrund

Die Einschätzung der Analgesie während der Anästhesie beruht (weiterhin) überwiegend auf der Interpretation klinischer Parameter wie Blutdruck, Herzfrequenz oder Tränenfluss [7]. Diese sind jedoch weder sehr sensitiv noch spezifisch, da auch andere Ursachen, wie Hypovolämie, geringe Narkosetiefe und bestimmte Medikamente, zu einer Veränderung dieser Parameter führen können.

Dies kann leicht zu einer Über-, aber auch Unterdosierung von Analgetika mit entsprechenden Nebenwirkungen (z. B Hypertension, Tachykardie, Koronarischämie, verstärkte postoperative Schmerzen bei Unterdosierung einerseits, Hypotonie, Bradykardie, Opioidüberhang, Übelkeit und Erbrechen bei Überdosierung andererseits) führen. Als Folge dieser Nebenwirkungen könnte es letztlich zu einer verzögerten Rekonvaleszenz, Steigerung von Morbidität und Mortalität der Patienten kommen [1]. Somit wird klar, dass eine adäquate intraoperative Analgesie auch für den weiteren postoperativen Aufenthalt im Aufwachraum (AWR) und auf Station bedeutsam ist und einen erheblichen Beitrag zur Patientenzufriedenheit leisten könnte.

Ein für die Nozizeption objektives und kontinuierliches Messverfahren könnte eine bedarfsgerechtere intraoperative Analgetikadosierung ermöglichen und die genannten Komplikationen reduzieren.

Ein solches nichtinvasives und leicht anzuwendendes Messverfahren soll der „Analgesia Nociception Index“ (ANI) bieten. Hierbei wird durch Analyse des EKG-Signals die Herzfrequenzvariabilität (HRV) ermittelt, welche Rückschlüsse auf die Balance zwischen Sympathikus- und Parasympathikusaktivität zulässt. Dies ermöglicht eine Aussage über den aktuellen Stresslevel des Patienten und damit (indirekt) über seine Nozizeption. Im Gegensatz zur subjektiven „Schmerzempfindung“ stellt der Begriff der „Nozizeption“ die neuronale Verarbeitung eines schmerzhaften Stimulus und die darauffolgende Reaktion des autonomen Nervensystems dar und unterliegt damit nicht der bewussten Wahrnehmung. Diese Nozizeption zu messen, ist und bleibt eine (medizinische) Herausforderung mit dem Ziel, postoperative Schmerz zu reduzieren und eine möglichst bedarfsgerechte Analgesie zu gewährleisten.

**Figure 1:**
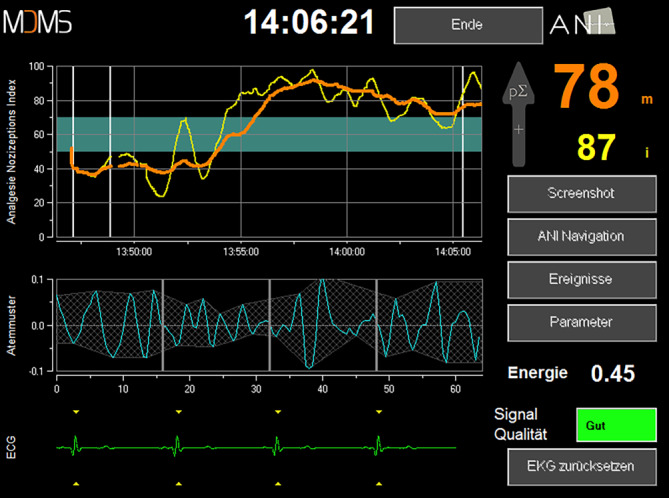

Angesichts divergierender Ergebnisse von anderen Studien zu diesem Thema (u. a.[13, 14]) war es Ziel unserer Untersuchung zu klären, ob es unter Verwendung des Analgesia Nociception Index (ANI, Abb. 1) bei Allgemeinanästhesie zu einer Veränderung des intraoperativen Opioidverbrauchs (primäre Zielgröße) kommt, und ob dadurch eine Optimierung der intra- und perioperativen Schmerztherapie ermöglicht wird. Optimierung wurde dahingehend definiert, dass es durch eine individualisierte intraoperative Analgetikatherapie zu einer Reduzierung von postoperativen Schmerzen, einem geringeren Auftreten von opioidbedingten Nebenwirkungen und letztendlich einer höheren Zufriedenheit der Patienten kommt.

## Studiendesign

In der prospektiven randomisierten Single-Center-Einfachblindstudie wurde an Hysterektomiepatientinnen im Alter zwischen 30 und 80 Jahren untersucht, ob die Verwendung des ANI-Monitors (MDoloris Medical Systems, Loos, France) zur intraoperativen Analgesiesteuerung Einfluss auf den intraoperativen Opioidverbrauch hat (primäres Outcome). Daneben wurden Auswirkungen bezüglich postoperativer Schmerzen, opioidinduzierter Nebenwirkungen (Atemnot, Übelkeit und Erbrechen, Vigilanzminderung) und Patientenzufriedenheit mit der Schmerztherapie untersucht (sekundäre Outcomes).

Die Rekrutierung der Patientinnen und die Durchführung der Studie erfolgten an der AGAPLESION DIAKONIE KLINIKEN KASSEL gGmbH in der Abteilung für Anästhesie und Intensivmedizin in Zusammenarbeit mit der Abteilung für Gynäkologie und Geburtshilfe im Zeitraum 06.01.2020 bis 24.07.2020.

Ein positives Ethikkommisionsvotum (FF 19/2019) der Landesärztekammer Hessen vom 20.12.2019 liegt vor. Es erfolgte eine Registrierung im Deutschen Register Klinischer Studien (DKRS-ID 00016965).

Eingeschlossen wurden Patientinnen mit einer Indikation zur laparoskopischen Hysterektomie (ASA1–2). Ausschlusskriterien waren z. B. Medikamentenunverträglichkeiten, ASA > 2, Vorerkrankungen oder Medikamenteneinnahmen, welche die Messungen beeinflussen könnten (z. B. Diabetes mellitus, Herzrhythmusstörungen, Einnahme von β‑Blockern).

Vor Beginn der Studie wurde eine formale Fallzahlschätzung anhand publizierter Metaanalysen von Grünewald [5] und Won [15] durchgeführt. Basierend auf diesen Studien wurde für die Fallzahlabschätzung eine Effektgröße von d = 0,5 für die Reduktion des intraoperativen Opioidverbrauchs angenommen. Hiermit ergab sich mit einem α = 0,05 und einem 1 − β = 0,80 eine notwendige Fallzahl von 100 Patienten.

Nach Aufklärung und schriftlichem Einverständnis wurden die Patientinnen vor der Operation in 2 homogene Gruppen randomisiert. Die Randomisierung erfolgte mittels Software des Research Randomizer (Urbaniak, G. C., & Plous, www.randomizer.org). Die Teilnehmerinnen waren bezüglich ihrer Gruppenzugehörigkeit verblindet.

Die Narkoseeinleitung erfolgte gewichtsadaptiert („lean body weight“ [LBW]) mittels Propofol, Fentanyl und Atracurium zur Muskelrelaxation. Die Patientinnen erhielten eine standardisierte PONV-Prophylaxe mit jeweils 4 mg Dexamethason und Ondansetron. Die Narkose wurde mittels Sevofluran EEG-gesteuert aufrechterhalten. In beiden Gruppen wurde kontinuierlich der ANI über eine zusätzlich am Thorax platzierte Doppelelektrode während des gesamten Narkoseverlaufs aufgezeichnet. So konnte auch in der Vergleichsgruppe detektiert werden, wie lange sich der ANI-Wert unterhalb des optimalen Bereichs (ANItime < 50 [%]) befand und mit der Interventionsgruppe verglichen werden. In der Interventionsgruppe (ANI) war der Indexwert für den Anästhesisten jederzeit sichtbar, und die Analgetikatherapie erfolgte anhand dieses Wertes. In der Vergleichsgruppe (VER) war der Index hingegen nicht einsehbar, und die Analgetikatherapie erfolgte nach Einschätzung des Anästhesisten anhand klinischer Vitalparameter (Blutdruck, Herzfrequenz). Die Analgetikatherapie erfolgte in beiden Fällen jedoch immer innerhalb der zulässigen Dosierungsgrenzen laut Fachinformation. Die Bolusgröße an Fentanyl in beiden Gruppen betrug jeweils 0,1 mg und war somit (anders als die Einleitungsdosis) nicht gewichtsadaptiert.

Verwendet wurde der Analgesia-Nociception-Index(ANI)-Monitor der Fa. MDoloris (Medical Systems, Loos, France) [4, 6–8].

Hierbei erfolgt die Messung anhand der Analyse der Herzfrequenzvariabilität in Abhängigkeit von der Atmung. Angegeben wird der Index in Form eines dimensionslosen Scores zwischen 0 und 100, wobei 0 für eine fehlende parasympathische Aktivität und 100 für eine sehr starke parasympathische Aktivität steht. Laut Hersteller entspricht ein Wert zwischen 50 und 70 in Narkose einer ausreichenden intraoperativen Analgesie. Eine Fentanylbolusgabe erfolgte dementsprechend bei einem ANI-Wert < 50. Der Screenshot des ANI-Monitors in Abb. 1 zeigt die ermittelten Messwerte unter Allgemeinanästhesie in graphischer und numerischer Form.

Neben dem Standard-Monitoring (Blutdruck nach RR, EKG, S_p_O_2_ über Fingerclipsensor) erfolgte die Messung der Narkosetiefe mittels Bispektralindex (BIS-MonitorTM der Firma Medtronic, Boulder, CO, USA), mit einem Zielwert von 40 bis 60.

Bolusanzahl und Gesamtmenge an Opioidverbrauch wurden dokumentiert.

Postoperativ wurden im Aufwachraum alle 15 min ein Schmerzscore mittels der numerischen Rating-Skala (NRS; 0–10) sowie opioidinduzierte Nebenwirkungen und Schmerzmittelverbrauch auf einem Studienerhebungsbogen dokumentiert. Ab einer NRS > 4 wurden 3–4 mg Piritramid i.v. verabreicht. Im Falle von Shivering wurde ein Clonidinbolus und im Falle von PONV 31–62 mg Dimenhydrinat i.v. verabreicht.

Die standardisierte postoperative Schmerztherapie auf der Station beinhaltete als Basismedikation Ibuprofen 4‑mal 400 mg p.o., Novaminsulfon 4‑mal 1 g i.v. als Kurzinfusion oder als Tropfen, Oxycodon 2‑mal 10 mg p.o. und als Bedarfsmedikation Piritramid 7,5 mg als Kurzinfusion. Am dritten postoperativen Tag wurden die Patientinnen auf der Station mithilfe eines hierfür angefertigten Fragebogens bezüglich Nebenwirkungen, Schmerzen und Gesamtzufriedenheit befragt.

Die Daten beider Gruppen wurden anschließend ausgewertet und bezüglich intraoperativem Gesamtverbrauch an Fentanyl, Schmerzscore (NRS) und Nebenwirkungen im Aufwachraum sowie Patientenzufriedenheit verglichen.

Ziel der Studie war es zu überprüfen, ob die Verwendung des ANI-Monitors, im Vergleich zum üblichen klinischen Standard, zu einer Optimierung der individuellen Analgetikatherapie bei Patientinnen mit laparoskopischer Hysterektomie unter balancierter Anästhesie mit Sevofluran führt.

### Statistik

Demografische und operative Daten werden tabellarisch und grafisch dargestellt.

Kontinuierliche Variablen werden als Mittelwert ± Standardabweichung angegeben.

Kategorische Daten werden als Anzahl und Prozentsatz der Grundgesamtheit angegeben.

Bei der Angabe des Mittelwerts der Anzahl kategorischer Merkmale wurde zudem die Spannweite (d. h. kleinste und größte Anzahl des Merkmals) angegeben.

Normalität der Verteilung wurde anhand des Shapiro-Wilk-Test geprüft.

Für normalverteilte Daten wurde anschließend der zweiseitige *t*-Test verwendet, andernfalls wurde der Mann-Whitney-U-Test angewendet.

Für kategorische Daten wurde der Chi-Quadrat-Test verwendet.

Eine multivariate lineare Analyse wurde anhand eines verallgemeinerten linearen Modells (GLM) durchgeführt.

Das lineare Regressionsmodell untersuchte dabei den möglichen linearen Zusammenhang zwischen der Zielgröße (z. B. die Fentanylmenge) der Gruppenvariable (ANI oder VER Gruppe) sowie möglichen Störvariablen (z. B. Alter, Gewicht, Operationsdauer). Ein *p*-Wert von 0,05 wurde als Signifikanzniveau verwendet.

## Ergebnisse

### Demografische Daten

**Figure 2:**
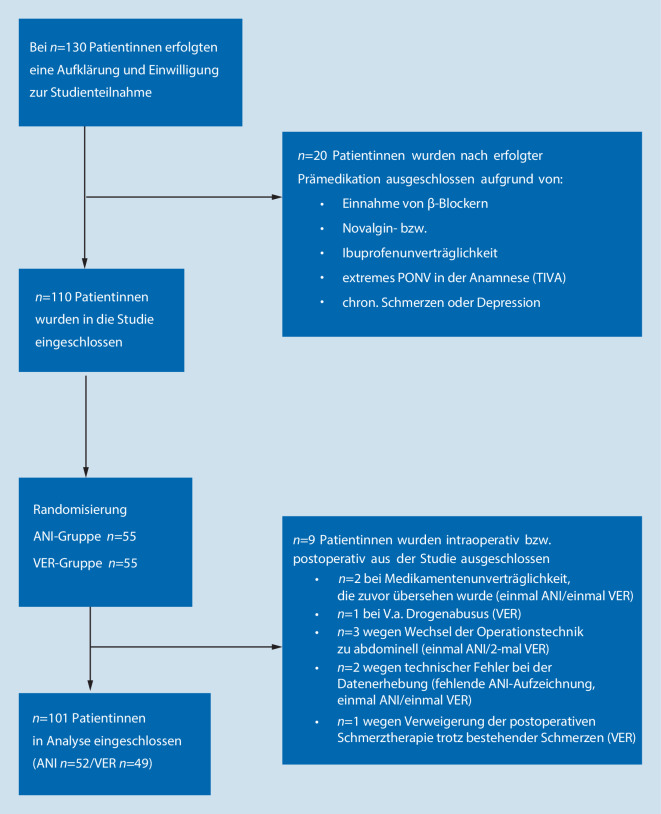

Insgesamt 130 Patientinnen, welche sich einer laparoskopischen Hysterektomie in unserer Einrichtung unterzogen, wurden in die Studie eingeschlossen. 20 Patientinnen wurden nach erfolgter Aufklärung und Prämedikation aufgrund eines oder mehrerer Ausschlusskriterien von der Teilnahme ausgeschlossen. Schließlich wurden 110 Patientinnen (ANI = 55, VER = 55) in die Studie aufgenommen. Postoperativ wurden weitere 9 Patientinnen ausgeschlossen (siehe Abb. 2 „Flow-Chart Randomisierung“). In die Auswertung flossen somit die Daten von 101 Patientinnen ein (ANI = 52, VER = 49). Siehe hierzu das Consort-Diagramm in Abb. 2.

Das Durchschnittsalter der Patientinnen betrug 48 ± 9 (33 bis 78) Jahre und unterschied sich nicht signifikant zwischen den Gruppen. Auch bezüglich des Gewichts und der Körpergröße gab es keinen signifikanten Unterschied zwischen den beiden Gruppen (Tab. 1).

**Table 1:** 

	Insgesamt	ANI-Gruppe	VER-Gruppe	*p*-Wert
**Demografie**	*N* = 101	*N* = 52	*N* = 49	–
Alter, Jahre (min/max)	48 ± 9 (33/78)	48 ± 9 (33/75)	48 ± 8 (35/78)	0,97
Körpergröße, cm (min/max)	167 ± 7 (153/186)	166 ± 8 (153/186)	167 ± 6 (156/177)	0,52
Gewicht, kg (min/max)	77 ± 18 (45/137)	79 ± 21 (49/137)	74 ± 15 (45/114)	0,15
**Operative Daten**				
Operationsdauer, min (min/max)	79 ± 31 (40/230)	84 ± 31 (40/190)	75 ± 31 (40/230)	0,19
**Operationstechnik**				
LASH TLH	48 (47,5 %) 53 (52,3 %)	29 (53,8 %) 24 (46,2 %)	19 (40,8 %) 29 (59,2 %)	0,16

*LASH* laparoskopische suprazervikale Hysterektomie, *TLH* totale laparoskopische Hysterektomie, *min* Minimum, *max* Maximum

Alle Patientinnen erhielten eine laparoskopische Hysterektomie, welche entweder als totale laparoskopische Hysterektomie (TLH) oder als laparoskopische suprazervikale Hysterektomie (LASH) durchgeführt wurde. Der Unterschied in der Verteilung beider Verfahren war statistisch nicht signifikant. Die beiden Gruppen waren in ihrer Zusammensetzung homogen und daher gut vergleichbar.

### Intraoperativer Fentanylverbrauch

Primäre Zielgröße war der intraoperative Opioidverbrauch (Fentanyl) im Vergleich zwischen Interventions- und Vergleichsgruppe. Die bei allen Operationen insgesamt verabreichte Fentanylmenge betrug 49 mg (ANI: 28 mg (57 %); VER: 21 mg (43 %); *p* < 0,001). Die durchschnittliche Fentanylgabe pro Operation betrug insgesamt 0,49 (0,2–0,7) mg (ANI: 0,54 (0,2–0,7) mg; VER:0,44 (0,3–0,7) mg; *p* < 0,001).

Minimal wurden 0,25 mg und maximal 0,7 mg Fentanyl während einer Operation verabreicht. Die durchschnittliche Bolusgröße betrug 0,1 mg in beiden Gruppen. Innerhalb aller Operationen wurden insgesamt 243 Boli verabreicht (ANI: 152 (62,5 %), VER: 91 (37,5 %) *p* < 0,001). Die durchschnittliche Anzahl der Boli betrug 2,4 (ANI: 2,9 (0–8), VER: 1,86 (0–5); *p* < 0,001).

**Figure 3:**
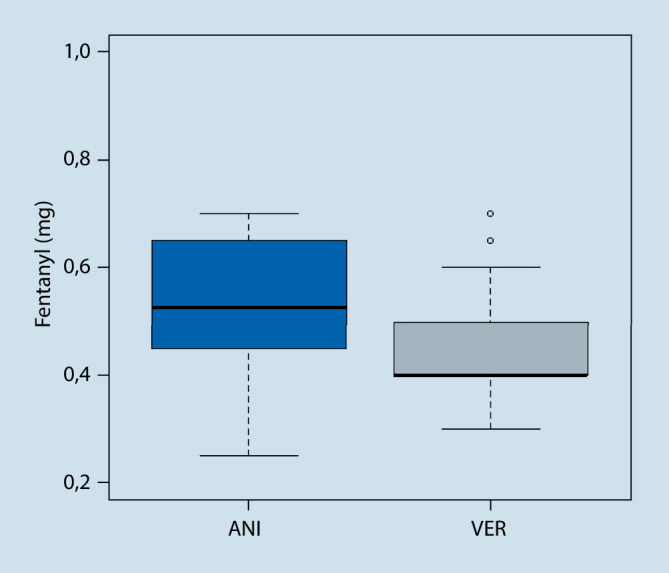

Demnach wurden in der Interventionsgruppe (ANI-Gruppe) statistisch signifikant mehr Fentanylboli und eine höhere Fentanylgesamtdosis verabreicht als in der Vergleichsgruppe (VER-Gruppe). Der Box-Plot in Abb. 3 gibt hierzu eine grafische Darstellung.

Zum Ausschluss möglicher Confounder wurde zudem geschaut, ob ein Zusammenhang zwischen den verabreichten Fentanylmengen und möglichen Einflussfaktoren wie Alter oder Gewicht der Patientinnen sowie der Operationsdauer besteht. In einer multivariaten Analyse waren sowohl das Gewicht, das Alter wie auch die Operationsdauer signifikant mit der Fentanylmenge assoziiert. Nach Kontrolle der Faktoren ergaben sich Fentanylmengen von 0,54 ± 0,06 mg in der ANI-Gruppe und von 0,44 ± 0,06 mg in der Vergleichsgruppe. Der Unterschied ist nach Kontrolle weiterhin signifikant (*p* < 0,001).

Des Weiteren wurde geschaut, inwieweit sich der mittlere ANI-Wert in beiden Gruppen unterscheidet, und wie lange sich dieser Wert prozentual unterhalb des optimalen Bereiches (ANI < 50) befand. In der ANI-Gruppe lag der durchschnittliche ANI-Wert bei 59 ± 10 und der Operationszeitanteil mit einem ANI-Wert < 50 bei 35 %. In der VER-Gruppe betrug der durchschnittliche ANI-Wert 57 ± 10 und die ANItime < 50 betrug 32 %. Es war somit kein signifikanter Unterschied zwischen den beiden Gruppen feststellbar, gleichwohl man ein geringeren Wert für ANItime < 50 in der ANI-Gruppe hätte erwarten können (*p* = 0,399).

### Schmerzscore im Aufwachraum

**Figure 4:**
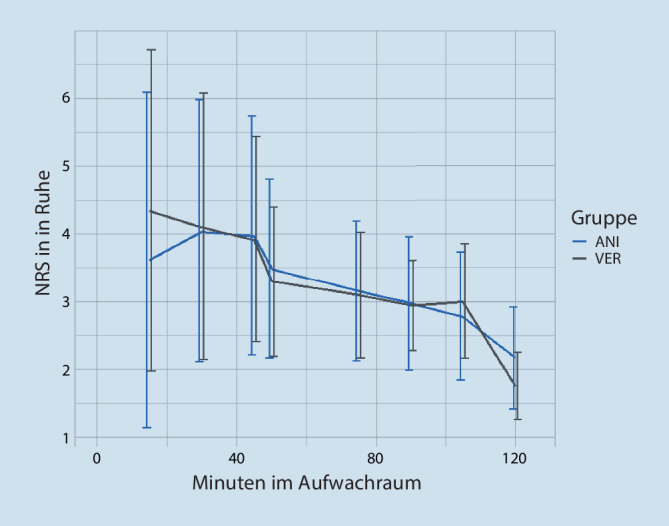

Beim Vergleich der Mittelwerte der erhobenen NRS-Werte (in Ruhe) zeigt sich kein signifikanter Unterschied zwischen den beiden Gruppen. Allenfalls bestand zum ersten Messzeitpunkt im AWR (NRS 15 min) ein Trend zu einem geringfügig niedrigeren Schmerzscore (3,6 ± 2,5 vs. 4,3 ± 2,4, *p* = 0,149). Hier zeigten die Patientinnen der ANI-Gruppe eine 88 % höhere Wahrscheinlichkeit für einen niedrigeren NRS als die Patientinnen der Vergleichsgruppe. Dieser Messzeitpunkt verpasst nur knapp die statistische Signifikanz (Abb. 4).

### Nebenwirkungen im Aufwachraum

Die Tab. 2 listet die erhobenen Nebenwirkungen in ihrer Häufigkeit (absolut und in Prozentanteil) im Gruppenvergleich auf.

**Table 2:** 

Nebenwirkung	Gesamt (*n* = 101)	ANI-Gruppe (*n* = 52)	VER-Gruppe (*n* = 49)	*p*-Wert
PONV	11 (11 %)	8 (15 %)	3 (6 %)	0,24
Vigilanzminderung	16 (16 %)	9 (17 %)	7 (14 %)	0,88
Luftnot	5 (5 %)	4 (8 %)	1 (2 %)	0,41
Clonidin/Shivering	15 (15 %)	9 (17 %)	6 (12 %)	0,66

*PONV* postoperative Übelkeit und Erbrechen („postoperative nausea and vomiting“)

Insgesamt konnte die Nebenwirkung „Übelkeit“ bei 11 Patientinnen während ihres Aufenthalts im Aufwachraum beobachtet werden. Dies entspricht 10,9 % der gesamten Fälle. 8 der 11 Fälle traten dabei in der ANI-Gruppe auf (entspricht 73 % der beobachteten Fälle) und der Rest in der Vergleichsgruppe (entspricht 27 % der beobachteten Fälle). Auch die anderen beobachteten Nebenwirkungen wie Vigilanzminderung oder Luftnot waren etwas häufiger in der ANI-Gruppe zu finden (ohne signifikanten Unterschied).

### Patientenzufriedenheit und Nebenwirkungen am 3. postoperativen Tag

Auch die Patientenzufriedenheit mit der Schmerztherapie zeigte keinen signifikanten Unterschied zwischen den Gruppen (Tab. 3) „Postoperative Übelkeit und Erbrechen“ (PONV) während der ersten 3 postoperativen Tage trat insgesamt bei 32,5 % der Patientinnen auf und war auf beide Studiengruppen gleichmäßig verteilt.

**Table 3:** 

	Gesamt (*n* = 80)	ANI-Gruppe (*n* = 41)	VER-Gruppe (*n* = 39)	*p*-Wert
Zufriedenheit (min–max)	1,6 ± 0,9 (1–5)	1,73 ± 1,0 (1–5)	1,45 ± 0,7 (1–4)	0,196

„Vigilanzminderung“, im Sinne von starker Müdigkeit, Einschränkung der Körperkontrolle, Orientierung und Kommunikationsfähigkeit, wurde von insgesamt 11 Patientinnen berichtet (13,3 %), wobei 23,3 % der Fälle in der ANI-Gruppe und 2,5 % der Fälle in der Vergleichsgruppe auftraten. Der Chi-Quadrat-Test nach Pearson ergibt hierfür einen Wert von 0,01 und liegt somit im signifikanten Bereich. Die anderen Nebenwirkungen unterschieden sich in diesem Zeitraum nicht.

„Luftnot“ (Einschränkung beim Schlucken und/oder Husten, subjektive Luftnot oder O_2_-Therapie auf der Station) betraf insgesamt 14,5 % der Patientinnen, 20,9 % der ANI-Gruppe und 7,5 % der Vergleichsgruppe.

Als häufigste Nebenwirkung traten Verdauungsprobleme in beiden Gruppen annähernd mit gleicher Häufigkeit auf. Auch Schmerzen trotz Schmerztherapie waren mit 37,3 % der Fälle relativ häufig und auf beide Gruppen gleich verteilt.

## Zusammenfassung der Ergebnisse

Intraoperativ zeigte sich ein erhöhter Gesamtverbrauch an Fentanyl in der ANI-Gruppe als Folge einer vermehrten Boligabe (0,1 mg Fentanyl/Bolus). Postoperativ zeigte sich im Aufwachraum kein signifikanter Unterschied zwischen den Gruppen hinsichtlich Schmerzscore, Opioidverbrauch oder opioidinduzierter Nebenwirkungen. Am 3. postoperativen Tag fand sich ein statistisch signifikanter Unterschied bezüglich der Vigilanzminderung, jedoch nicht bei den anderen beobachteten Nebenwirkungen oder mit Blick auf die Patientenzufriedenheit.

### Schlussfolgerung

Die höhere intraoperative Fentanyldosis wäre, für sich betrachtet, mit einer bedarfsgerechteren Opioidtherapie vereinbar. Die im Aufwachraum und auf der Station nichtnachgewiesenen Unterschiede im Bereich von Nebenwirkungen und Patientenzufriedenheit lassen in unserer Studie jedoch nicht auf einen sichtbaren Vorteil einer ANI-gesteuerten intraoperativen Analgetikadosierung schließen (bezogen auf das vorliegende Studienkollektiv).

## Diskussion

### Intraoperativer Fentanylverbrauch

Bei der statistischen Auswertung der Studienergebnisse ließ sich ein signifikanter Unterschied im Fentanylverbrauch zwischen den zu vergleichenden Gruppen feststellen. Der in der Interventionsgruppe (ANI-Gruppe) signifikant höhere Fentanylverbrauch ergab sich durch die höhere Anzahl an Fentanylboli.

Der die Narkose durchführende Anästhesist wurde demnach durch die Verwendung des ANI dazu veranlasst, im Operationsverlauf mehr bzw. häufiger Fentanyl zu applizieren, als er es ohne Zuhilfenahme des ANI getan hätte.

Die maximal verabreichte Bolusanzahl betrug in der ANI-Gruppe 8, im Vergleich zu 5 in der Vergleichsgruppe. Die Bolusgabe erfolgte bei einem Wert ANI < 50. Blieb der gewünschte Erfolg aus (Anstieg > 50), so wurde nach 4–6 min (erwarteter maximaler Wirkungseintritt für Fentanyl) erneut ein Bolus gegeben.

In einer vergleichbaren Studie aus dem Jahr 2017 von Upton et al. mit dem postoperativen Schmerz als primärem Endpunkt zeigte sich dagegen eine etwas geringere intraoperative Gesamtdosis für Fentanyl in der Interventionsgruppe (ANI-Gruppe), wobei die Anzahl der Boli ebenfalls erhöht, die Bolusgröße jedoch gegenüber der Vergleichsgruppe reduziert war. Daraus wurde geschlussfolgert, dass die Dosierung von Analgetika mit ANI bedarfsgerechter erfolgen kann und zu einer Reduzierung von postoperativen Schmerzen und des Schmerzmittelbedarfs im AWR führt [14]. Diese Studienergebnisse bestätigen demnach unsere eigene Beobachtung von vermehrter Boligabe unter ANI-Monitoring, jedoch nicht die von uns beobachtete erhöhte Gesamtdosis und den bei uns fehlenden Unterschied beim Schmerzscore im Aufwachraum.

Eine andere Studie an 120 Patienten mit Cholezystektomie, wobei intraoperativ Morphin als Opioid verwendet wurde, konnte dagegen keinen Unterschied zur Vergleichsgruppe im Gesamtverbrauch feststellen [13]. Die Unterschiede der Ergebnisse sind u. U. auf die unterschiedliche Pharmakokinetik der verwendeten Opioide oder auch auf die verschiedenen Operationsformen (Schmerzqualität) zurückzuführen. Ebenso wird in der Studie von Szental et al. das Pneumoperitoneum durch dessen Einfluss auf den vagalen Tonus mit konsekutiver Veränderung von Blutdruck und Herzfrequenz als mögliche Limitierung für die ANI-Messung benannt [13], welches auch bei uns Bestandteil jeder Operation war. In diesem Zusammenhang ist durchaus zu diskutieren, ob eine andere Operationsform oder die Verwendung eines Nozizeption-Monitors, dessen Messung sich nicht ausschließlich auf den Vagustonus stützt (z. B. Nociception Level [NOL] Index), eine bessere Wahl für das vorliegende Studiendesign gewesen wäre.

Weitere Studien, wie z. B. von Deccache et al. von 2016, verwendeten Remifentanil zur intraoperativen Analgesiesteuerung und kamen dabei zu dem Schluss, dass sich dieses durch Verwendung des ANI bedarfsgerechter dosieren und unerwünschte hämodynamische Ereignisse sowie Opioidverbrauch und postoperative Schmerzen reduzieren lassen [3]. Zu ähnlichen Ergebnissen kam eine Studie in der Adipositaschirurgie unter TIVA [10]. Nicht auszuschließen ist eine effektivere Steuerung mit ANI unter Verwendung eines kurz wirksameren Opioids mit schnellerem Wirkeintritt, wie z. B. bei TIVA der Fall. Insgesamt bleiben die Studienergebnisse jedoch divergent.

Vergleichbar mit unsrigen Ergebnissen, fand auch die Studie von Szental et al. [13] keinen Unterschied in der Häufigkeit bzw. Zeitdauer, bei der die Probanden einen ANI-Wert < 50 hatten. Anzunehmen wäre eine geringere Anzahl von Probanden mit einem ANI < 50 in der ANI-Gruppe. Eine mögliche Erklärung dafür könnte auch in diesem Fall die Auswahl des Opioids und dessen Applikationsform sein.

### Schmerzscore und Nebenwirkungen im Aufwachraum

Bezüglich der Endpunkte Schmerzscores im AWR, Nebenwirkungen und Schmerzmittelverbrauch zeigte sich kein signifikanter Unterschied zwischen den beiden Gruppen. Die Dosierung der Analgetika anhand des ANI hatte somit keinen Einfluss auf das direkte postoperative Wohlbefinden oder das Auftreten von opioidinduzierten Nebenwirkungen, obwohl die Patientinnen der ANI-Gruppe im Durchschnitt intraoperativ mehr Fentanyl erhalten hatten.

Lediglich der NRS zum ersten Messzeitpunkt (15 min) nach Eintreffen im AWR lässt einen Trend dahingehend erkennen, dass die Patientinnen der ANI-Gruppe im Durchschnitt einen geringeren Wert angaben. Dieser Unterschied ist zwar sichtbar, verfehlt jedoch knapp die Signifikanzgrenze und ist hinsichtlich der Größe (< 1 Skalenpunkt) von fraglicher klinischer Relevanz. Er könnte jedoch ein Hinweis darauf sein, dass die Patientinnen der ANI-Gruppe zumindest direkt postoperativ von einer geringeren Schmerzintensität profitierten. Allerdings gab es ebenso einen Trend zu häufigeren opioidbedingten Nebenwirkungen wie PONV.

Ebenso bedeutsam könnte sein, dass „Schmerz“ (im Gegensatz zur Nozizeption) von vielen Faktoren beeinflusst wird. So auch von Ängsten und Erwartungen des Patienten. So bleibt die numerische Rating-Skala eine rein subjektive Messung, die besser für die Verlaufskontrolle bei einem Patienten als für den Vergleich zwischen mehreren Patienten bzw. Gruppen untereinander geeignet ist. Dennoch bleibt sie ein bewehrtes und viel eingesetztes Messinstrument in Studien.

Es ist zu vermuten, dass die Umstände im AWR (Lärm, ungewohnte Umgebung etc.) und die individuellen Zustände des Patienten (Erregung, Stress, Angst, Persönlichkeitsmerkmale, Alter) einen solch starken Einfluss ausüben, dass die intraoperative Schmerztherapie eine hierbei eher untergeordnete Rolle spielt und daher keinen signifikanten Unterschied im Schmerzscore erkennen lässt.

### Patientenzufriedenheit am 3. postoperativen Tag

Soweit bekannt, handelt es sich bei unserer Studie um die erste, die sich mit der Frage der Patientenzufriedenheit mit der Schmerztherapie unter intraoperativer Verwendung eines Nozizeption-Monitorings befasst. Daher liegen hierzu keine vergleichenden Daten vor. Allerdings findet sich in einer US-amerikanischen Studie zur generellen Inzidenz von postoperativen Schmerzen auch der Aspekt der Patientenzufriedenheit. In der Studie wurde mit einem vergleichbaren Fragebogen wie dem unsrigen gearbeitet. Bei sinkender Schmerzintensität stieg hier zwar die Patientenzufriedenheit, dennoch erschien die Patientenzufriedenheit im Vergleich zu der bei Entlassung noch relativ hohen Schmerzintensität überdurchschnittlich hoch [2].

Es wurde ein Fragebogen zur Erfassung von opioidinduzierten Nebenwirkungen und der Patientenzufriedenheit entworfen, welchen die Probanden am 3. postoperativen Tag auf der Station beantworteten (Zusatzmaterial online). Der Fragebogen orientierte sich an dem „Medlinq-Patientenfragebogen“ des von uns verwendeten Narkoseprotokolls von „Medlinq 2018“ und behandelte die Themenbereiche „postoperative Übelkeit und Erbrechen“ (PONV), „Schmerzen“, „Verdauungsprobleme“, „Atembeschwerden“ und „Vigilanz“, aber auch die Zufriedenheit mit der Betreuung auf der Station, im Sinne einer ausreichenden Beachtung geäußerter Beschwerden [12]. Zusätzlich sollte seitens der Patientinnen eine Bewertung in Form von Noten (*1* sehr gut bis *6* ungenügend) bezüglich der Gesamtzufriedenheit mit der Schmerztherapie vergeben werden.

Bei der Auswertung der Fragebogen fiel auf, dass die Vergabe der Noten scheinbar unabhängig vom Auftreten der Nebenwirkungen und der postoperativ empfundenen Schmerzen war. So vergaben Patientinnen, welche das Auftreten von sämtlichen Nebenwirkungen verneinten, und die postoperativen Schmerzen als gering bewerteten, z. T. schlechtere Gesamtnoten als Patientinnen, welche z. B. unter starker Übelkeit und stärkeren Schmerzen zu leiden hatten. Dies macht deutlich, dass auch die Bewertung einer stattgefundenen Behandlung (ähnlich wie die Schmerzwahrnehmung selbst) von vielen Faktoren beeinflusst wird und stark vom subjektiven Empfinden des Befragten abhängt. So wurden die Schmerzen als deutlich erträglicher empfunden, wenn sie durch das Pflegepersonal entsprechende Beachtung fanden.

Unsere Ergebnisse stehen somit im Einklang mit den Beobachtungen der oben zitierten Studie, welche die Ergebnisse mit der Erwartungshaltung der Patienten erklärt. Das bedeutet, ein gewisser Anteil an Schmerz nach einer Operation wurde von den Patienten erwartet und hatte daher kaum negativen Einfluss auf die Patientenzufriedenheit. Dies könne sich jedoch laut Autoren mit zukünftig wahrscheinlich steigender Erwartungshaltung bezüglich Schmerzfreiheit ändern und zukünftig eine Reduzierung der Zufriedenheit zur Folge haben. Daher sei eine Optimierung der bedarfsgerechten Analgesie auch im Hinblick auf die Patientenzufriedenheit von zunehmender Bedeutung [2].

Zwar wurden die Studienteilnehmerinnen untereinander von dem betreuenden Personal gleich behandelt (Verblindung), doch ist anzunehmen, dass sie im Vergleich zu Patienten ohne Studienteilnahme eine höhere Aufmerksamkeit erfuhren und dies insgesamt zu einer positiven Bewertung führte.

Zusätzlich ist es denkbar, dass allein die Information über die Teilnahme an einer Studie, welche eine Optimierung der Schmerztherapie zum Ziel hatte, zu einer gesteigerten Erwartungshaltung in diesem Bereich führte (unabhängig von der Gruppenzugehörigkeit). Es ist so nicht auszuschließen, dass (wenn auch unbewusst) tendenziell bessere Noten vergeben wurden, um den Erwartungen der Studienleitung zu entsprechen.

### Nebenwirkungen auf der Station

Bezüglich der Nebenwirkungen auf der Station fand sich kein Unterschied bei der Häufigkeit im Auftreten von Übelkeit und Erbrechen. Diese waren mit 67,4 % in der ANI-Gruppe gegenüber 67,5 % in der VER-Gruppe gleich häufig. Ebenso verhielt es sich mit den anderen Nebenwirkungen wie Schmerz, Atemnot und Verdauungsproblemen.

Anders hingegen bei der Vigilanz. Hier zeigte sich der einzige signifikante Unterschied zwischen den beiden Gruppen (*p* = 0,005). Eine Einschränkung der Vigilanz wurde insgesamt bei 11 Patientinnen (13,3 %) und davon 10-mal (23,3 %) in der ANI-Gruppe vs. einmal (2,5 %) in der VER-Gruppe beobachtet.

Es ist nicht auszuschließen, dass die vermehrte Fentanylgabe in der Interventionsgruppe (ANI-Gruppe) diesen nachteiligen (subjektiven) Effekt bewirkt hat. Dies hatte jedoch weder gesundheitliche Folgen noch einen Einfluss auf die Gesamtzufriedenheit der betroffenen Patientinnen, ist aber im Hinblick auf die frühe postoperative Mobilisation ein unerwünschter Effekt.

### Limitierungen der Studie

Mögliche Limitierungen der Studie finden sich u. a. im Studiendesign. So handelt es sich bei dem Patientenkollektiv um überwiegend jüngere weibliche Patientinnen ohne wesentliche Vorerkrankungen, ASA 1–2, welche keine Medikamente einnehmen, die einen Einfluss auf die Nozizeptionsmessung haben könnten. Fraglich bleibt daher, ob sich die von uns erhobenen Daten problemlos auf ein eher immer älter und kränker werdendes Patientenkollektiv übertragen lassen.

Auch ist die Operationsdauer mit durchschnittlich 79 min eher kurz. Es ist daher nicht auszuschließen, dass durch die standardisierte initiale Fentanyldosis bei Anästhesieeinleitung schon ein großer Teil des intraoperativen Opioidbedarfs abgedeckt wurde. Auch wäre zu überlegen, ob die Verwendung eines kürzer wirksamen Opioids mit schnellerem Wirkeintritt und somit besserer Steuerbarkeit zu anderen Ergebnissen geführt hätte.

Als einer der ersten publizierten Nozizeptionsindizes dient der ANI als Surrogatparameter für den Parasympathikotonus durch Analyse des auf der respiratorischen Arrhythmie beruhenden Variabilität des R‑R-Intervalls [11]. Über eine kontinuierliche Auswertung des EKG-Signals wird die Balance von Nozizeption und Analgesie bestimmt. Andere Verfahren nutzen hierfür u. a. die Hautleitfähigkeit, Hauttemperatur, EEG-Signale oder reizgetriggert den Pupillendilatations- oder Beugereflex. Ein Überblick über kommerziell erhältliche Systeme und deren Vor- und Nachteile findet sich in [9]. Vorteil des ANI ist seine einfache Anwendbarkeit durch Analyse des standardmäßig abgeleiteten EKG-Signals. Allerdings haben auch andere Effekte Einfluss auf die Messung. So machen z. B. eine Apnoe oder ein fehlender Sinusrhythmus eine Messung unmöglich, während verschiedene Medikamente und emotionaler Stress (bei wachen Patienten) eine Interpretation erschweren [11].

## Zusammenfassung

Ziel der vorliegenden randomisierten kontrollierten Einfachblindstudie war es zu untersuchen, ob die intraoperative Verwendung eines Analgesia Nociception Index (ANI) zur Analgesiesteuerung einen Einfluss auf den intraoperativen Opioidverbrauch (primäres Outcome), den postoperativen Schmerzscore, die opioidinduzierten Nebenwirkungen sowie auf die Patientenzufriedenheit mit der Schmerztherapie hat. Bei dem von uns gewählten Patientenkollektiv unter Verwendung von Fentanyl als Opioid ergab sich ein im Vergleich erhöhter Fentanylverbrauch durch vermehrte Boligabe. Kein signifikanter Unterschied zeigte sich im gemessenen Schmerzscore oder den Nebenwirkungen im Aufwachraum. Bei der Patientenbefragung am 3. postoperativen Tag zeigte sich ein signifikanter Unterschied bei der erhobenen Nebenwirkung „Vigilanzminderung“, jedoch ohne klinische Relevanz oder Einfluss auf die Patientenzufriedenheit.

Eine Optimierung der intraoperativen Schmerztherapie durch Zuhilfenahme des Analgesia-Nozizeption-Index(ANI)-Monitors bei Hysterektomiepatientinnen unter balancierter Anästhesie mit Sevofluran und Fentanyl konnte demzufolge nicht nachgewiesen werden.

## Fazit für die Praxis

- Der Einsatz des ANI zur intraoperativen Analgesiesteuerung bei laparoskopischen Hysterektomien unter balancierter Allgemeinanästhesie führte bei unserem Patientenkollektiv zu einem erhöhten Fentanylverbrauch.
- Es fand sich kein signifikanter Unterschied bezüglich postoperativer Schmerzen oder opioidinduzierter Nebenwirkungen.
- Die Patientenzufriedenheit war in beiden Gruppen vergleichbar.
- Die Übertragbarkeit der Studienergebnisse auf ein kränkeres und älteres Patientenkollektiv bleibt jedoch fraglich.

## Supplementary Information





## References

[1] Abdullayev R (2019). Analgesia Nociception Index: assessment of acute postoperative pain. Rev Bras Anestesiol.

[2] Buvanendran A, Fiala J, Patel KA, Golden AD, Moric M, Kroin JS (2015). The incidence and severity of postoperative pain following inpatient surgery. Pain Med.

[3] Daccache G (2016). A targetet remifentanil administration protocol based on the Analgesia Nociception Index during vascular sugery. Anaesth Crit Care Pain Med.

[4] Daccache G, Jeanne M, Fletcher D (2017). The Analgesia Nociception Index: Tailoring Opioid Administration. Int Anaesth Res. Soc.

[5] Gruenewald M, Willms S, Brach O, Kott M, Steinfath M, Bein B (2014). Sufentanil administration guided by surgical Plethi Index vs standard practice during sevoflurane anaesthesia: a randomized controlled pilot study. Br J Anaesth.

[6] Jeanne M, Delecroix M, De Jonckheere J, Keribedj A, Logier R, Tavernier B (2014). Variations of the Analgesia Nociception Index during Propofol anesthesia for total knee replacement. Clin J Pain.

[7] Jeanne M, Logier R, De Jonckheere J, Tavernier B (2009) Validation of a graphic measurement of heart rate variability to assess analgesia/nociception balance during general anesthesia. In International Conference of the IEEE EMBS, 2009 10.1109/IEMBS.2009.5332598 19963520

[8] Jeanne M, Tavernier B, Logier R, De Jonckheere J (2017). Closed-loop administration of general anaesthesia: from sensor to medical device. Pharm Technol. Hosp Pharm.

[9] Ledowski T (2019). Objective monitoring of nociception: a review of current commercial solutions. Br Jornal Anesth Pp.

[10] Le Gall L, David A, Carles P, Leuillet S, Chastel B, Fleureau C, Dewitte A, Ouattara A (2017). Benefits of intraoperative analgesia guided by the Analgesia Nociception Index (ANI) in bariatric surgery: an unmatched case-control study. SFAR.

[11] Nitzschke R, Fischer M, Funcke S (2021). Nozizeptionsmonitoring – Methode zur intraoperativen Opioidsteuerung?. Anaesthesist.

[12] qm-anaesthesie.de https://www.qm-anaesthesie.de/fachmaterial/downloads/ppp33-fragebogen/10-ppp33-fragebogen-version-050415-dina3/file.html. Zugegriffen: 1. Aug. 2019

[13] Szental J, Webb A, Weeraratne C, Campbell A, Sivakumar H, Leong S (2015). Postoperative pain after laparoscopic cholecystectomy is not reduced by intraoperative analgesia guided by Analgesia Nociception Index (ANI) monitoring: a randomized clinical trial. Brit J Anaesthes.

[14] Upton H, Ludbrook G, Wing A, Sleigh J (2017). „intraoperative „Analgesia Nociception Index“—guided fentanyl administration during sevoflurane anästhesie in lumbar discectomy and laminectomy: a randomized clinical trial. Anesth Analg.

[15] Won Y, Kim Y, Lee M, Kim H (2018). Usefulness of surgical pleats index—guided analgesia during general anesthesia: a systematic review and meta-analysis of randomized controlled trials. J Int Med Res.

