# Supplementary material for: Intraoperative guidance of anesthesia: Analgesie Nociception Index (ANI) vs. standard care for hysterectomy under anesthesia with sevoflurane: A randomized controlled simple blinded study on intraoperative opioid consumption, postoperative pain and patient satisfaction
Source: Anaesthesiologie. 2023 Jun 7;72(7):477–87. [Article in German] doi: 10.1007/s00101-023-01288-y (PMC10322764; doi:10.1007/s00101-023-01288-y)
Supplement: Supplementary file 1 [file 101_2023_1288_MOESM1_ESM.pdf]

Zusatzmaterial zum Beitrag **„Intraoperative Analgesiesteuerung: ANI versus Standard Care bei Hysterektomien unter Sevoflurannarkose“** von Kunst A, Wulf H, Stegmann B und Fiehn A (2023) in *Die Anaesthesiologie.*
Beitrag und Zusatzmaterial stehen Ihnen auf www.springermedizin.de zur Verfügung. Bitte geben Sie dort den Beitragstitel in die Suche ein.

Untersuchung zur Wertigkeit des Analgesia Nociception Index (ANI) bei Hysterektomien im Hinblick auf den intraoperativen und postoperativen Analgetika Bedarf

POSTOPERATIVER PATIENTENFRAGEBOGEN

Fragen	Trifft nicht zu	Trifft eher weniger zu	Trifft weitgehend zu	Trifft voll zu
Ich habe nach dem Eingriff schnell wieder die Kontrolle über meinen Körper erlangt				
Ich war nach dem Eingriff zu schwach, mich im Bett aufzurichten, richtig zu schlucken oder zu husten				
Ich war nach dem Eingriff schnell wieder orientiert und konnte mich gut verständlich äußern				
Ich hatte starke Schmerzen im Operationsgebiet				
Ich hatte starke Schmerzen an anderen Körperstellen (z.B. Kopf-, Hals-, Rücken-, Brust- oder Gelenkschmerzen)				
Meine Schmerzen waren außer Kontrolle und hätten noch besser behandelt werden müssen				
Ich hatte Probleme mit der Verdauung				
Ich habe Übelkeit empfunden oder musste erbrechen				
Meine Beschwerden wurden ausreichend beachtet und behandelt				
Wie zufrieden waren Sie insgesamt mit Ihrer Schmerztherapie?	**Bitte bewerten Sie diese mit einer „Schulnote“ zwischen 1 – 6**<br>1= sehr gut 2=gut 3=befriedigend 4=ausreichend 5=mangelhaft 6=ungenügend			

Wir bedanken uns für Ihre Unterstützung und wünschen Ihnen gute Besserung

In Anlehnung an Eberhard, Kranke, Simon , Celik © PPP33-Fragebogen

Untersuchung zur Wertigkeit des Analgesia Nociception Index (ANI) bei Hysterektomien im Hinblick auf den intraoperativen und postoperativen Analgetika Bedarf.

DATENBOGEN AUFWACHRAUM

Bitte erheben Sie bei den Patientinnen während des Aufenthaltes im Aufwachraum alle 15 Min. die NRS Werte in Ruhe / bei Bewegung und tragen diese in diesen Datenbogen ein. Weiterhin dokumentieren Sie bitte die verabreichte Menge der Analgetika sowie eventuell auftretenden Nebenwirkungen wie Übelkeit, Erbrechen, Vigilanz-Minderung, Atemnot. Sie helfen uns sehr damit, den Zusammenhang zwischen intraoperativer Analgetika Gabe, dem postoperativen Analgetika Verbrauch und den möglichen Nebenwirkungen besser zu verstehen. Ganz herzlichen Dank für diese zusätzlichen Mühen !

Patienten-Studien Nummer:

Aufnahme Uhrzeit im Aufwachraum		Messparameter						
	NRS Ruhe	NRS Bewegung	Piritramid in mg	Übelkeit	Erbrechen	Atemnot	Vigilanz Minderung	Sonstiges
15 Min.								
30 Min.								
45 Min.								
60 Min.								
75. Min								
90. Min								
105 Min.								
120 Min.								
135 Min.								
150 Min.								
165 Min.								
180 Min.								
195 Min.								
210 Min.								
225 Min.								
240 Min.								
